# Challenges and Results of Laparoscopic Splenectomy for Hematological Diseases in a Developing Country

**DOI:** 10.1155/2018/4256570

**Published:** 2018-08-01

**Authors:** Vikal Chandra Shakya, Bikram Byanjankar, Rabin Pandit, Anang Pangeni, Anir Ram Moh Shrestha, Bishesh Poudyal

**Affiliations:** ^1^Department of Surgery, Civil Service Hospital of Nepal, Kathmandu, Nepal; ^2^Clinical Hematology and Bone Marrow Transplant Unit, Department of Medicine, Civil Service Hospital of Nepal, Kathmandu, Nepal

## Abstract

**Introduction:**

Though, in developed countries, laparoscopy is now a gold standard for splenectomy, we are lacking in this aspect in the eastern world. Splenectomy has mostly been performed by open surgery in our region. This is our effort to introduce laparoscopic splenectomy in our country.

**Methods:**

This is a retrospective cohort study done in patients presenting to hematology and surgery department of our hospital who underwent laparoscopic splenectomy for hematological diseases from January 2013 to December 2016.

**Results:**

There were 50 patients (38 females, 12 males). The diagnoses were idiopathic thrombocytopenic purpura in 31, (steroid/azathioprine-resistant, steroid dependent), hereditary spherocytosis in 9, alpha-thalassemia in 3, beta-thalassemia in 2, autoimmune hemolytic anemia in 4, and isolated splenic tuberculosis in 1. Average platelet counts preoperatively were 62000 ± 11000/mm3 (range 52000-325000/mm3). The mean operative time was 130 ± 49 minutes (range 108-224 min). The mean postoperative stay was 4 ± 2.11 days (range 3-9 days). Laparoscopic splenectomy could be completed in 45 (90%) patients.

**Conclusion:**

Laparoscopic splenectomy could be successfully contemplated in patients with hematological diseases, especially if spleen is normal or only mildly enlarged, and is an advantageous alternative to open splenectomy. Absence of ideal resources has not limited our progress in minimal access approach.

## 1. Introduction

After the first report of laparoscopic splenectomy in 1991, this technique has been rapidly established to be a safe and effective treatment for a range of benign and malignant hematological conditions [1, 2]. It is associated with low morbidity rates of 18–26% [2–4] and zero mortality in some series [5–7], with others recently reporting mortality of up to 4% [2, 4, 8–12]. The hospital stay is also shorter [7, 13], and the conversion rate to open operation is reported at 0–15% in recent series [14–19]. Though laparoscopic splenectomy has been well accepted in the developed countries, advanced laparoscopic surgeries have been largely eluded in a country like ours. However, we have ventured to perform laparoscopic splenectomy in our country. The aim of this study was to evaluate the results of a single-center single-surgeon experience of laparoscopic splenectomy in patients with hematological diseases.

## 2. Methods

This is a retrospective cohort study done of prospective maintained data of all patients undergoing laparoscopic splenectomy for hematological diseases between January 2013 and December 2016 at Civil Service Hospital of Nepal, a government hospital at our capital Kathmandu, by a single surgeon. The methodology has been adapted from personal series by Paddenden et al. [20]. The data included patient age, hematological diagnosis, duration of operation, operative blood loss, size of spleen, conversion to open, postoperative complications, length of hospital stay, mortality, and the duration of follow-up.

Exclusion criteria were as follows:

1. Traumatic splenic injury
2. Splenectomy when combined with other operations as distal pancreatectomy, gastrectomy, and cholecystectomy
3. Massive splenomegaly: a spleen palpable inferior and to the right of the umbilicus
4. Preoperative low platelet counts <50000/ml.

The patients were also evaluated with radiological investigations to determine the size of the spleen, usually by ultrasonography, and computed tomography in case of large spleens and doubtful diagnosis. Vaccinations and antibiotic prophylaxis were given in accordance with the guidelines of the British Committee for Standards in Hematology; all patients were immunized against pneumococcus, hemophilus, and meningococcus at least 2 weeks preoperatively and influenza vaccine annually [21]. Antithrombotic prophylaxis was not given to patients with ITP which is an inherent bleeding disorder; in all other patients with normal platelet counts like in thalassemia, antithrombotic prophylaxis was given.

Patients were subjected to general anesthesia, paralyzed, and ventilated. Prophylactic antibiotic Ceftriaxone 1gm stat was given during induction of the anesthesia. They were positioned in a right, lateral, semisupine (60°) position [3]. A 10-mm umbilical port was inserted with an open technique using a blunt Hassan trocar at the midclavicular line. The remaining ports were inserted under direct vision. These consisted of three further ports, two in the left flank (10 mm and 5 mm ports), and one 5-mm port in the epigastrium, just inferior to the xiphoid process. A 30° scope was used in all cases. The spleens were mobilized with a combination of monopolar, bipolar cautery (Valleylab cautery machine), and ultrasonic energy source (Ethicon Harmonic, USA) when available. After opening the gastrocolic ligament by dividing the short gastric vessels, the hilar vessels and short gastric vessels were individually identified and divided between Hem-o-lok clips (Weg, UK). Staplers or mass ligation has not been used. The lienorenal ligament and lienophrenic ligament are divided with a combination of monopolar diathermy hook and scissors. The spleens were placed in endogenous bags made of urobags (Romo-10, India) with mouth sutured with retractable silk suture and morcellated intracorporeally with help of sponge-holding forceps, and Yankauer suction tip through the 10 mm port; and the spleen was retracted piecemeal through the midclavicular port with the urobag intact protecting the wound as well. Depending upon subjective assessment of the hemostasis, occasionally, 14-Fr suction drains (Romovac-Romson, India) were placed in the splenic beds. For large spleen, when the spleen was not possible to be kept in a bag and in which splenectomy was performed for diagnostic purposes, the spleen was delivered through transverse left lumbar or Pfannenstiel incision. The patients were transferred to a surgical postoperative ward for 4-hourly observations of pulse, blood pressure, and temperature. The following morning, the hemoglobin and platelet count were checked. The drains, if present, were removed provided the drainage was less than 50 ml over 24 hours. They were monitored for consequent hemoglobin and platelet increases daily and chest x-rays and ultrasound of the abdomen if any problems. Patients were discharged with oral analgesia once they were sufficiently comfortable, antibiotics for 7 days postoperatively, and their observations were satisfactory. The patients were seen once in general surgical outpatients to ensure good healing of wounds. The hematological follow-up depended upon the underlying diagnosis.

## 3. Results

During the study period, there were 50 consecutive laparoscopic splenectomies performed. There were 50 patients (38 females, 12 males). The mean age of presentation was 28 ± 10 years (range 14-56). The mean duration of symptoms was 4.65 ± 2.78 years (range 1.3-12.23 years). The average splenic size determined preoperatively was 13.45 ± 6.26 cm (range 10-23.5 cm). The diagnosis was Idiopathic Thrombocytopenic Purpura in 31 (steroid/azathioprine-resistant, steroid dependent), hereditary spherocytosis in 9, alpha-thalassemia in 3, beta-thalassemia in 2, autoimmune hemolytic anemia in 4, and isolated splenic TB in 1. Average platelet counts preoperatively was 62000 ± 11000/mm3 (range 52000-325000/mm3). The mean operative time was 130 ± 49 minutes (range 108-224 min) (Table 1). Average blood loss was 456 ml (from negligible to 1600 ml). Laparoscopic splenectomy could be completed in 45 (90%) patients. The remaining 5 (10%) needed conversion to open (causes being excessive bleeding from the splenic vein, splenic capsular tear and excessively low platelet counts with gross oozing) (Table 2). Five patients needed an additional Pfannenstiel or lumbar incision to retrieve the spleen after completion of procedure laparoscopically. Splenunculi (accessory spleens) were noted and excised from 3 patients in this series (6%). Postoperative morbidities were seen in 8 patients (16%). Postoperatively, one patient developed splenic and partial portal vein thrombosis and started on heparin and later warfarin; he was monitored with repeat Doppler ultrasonography of the abdomen; his vessels recanalized at 1.5 years of follow-up without any symptoms, and he is presently off warfarin. Packed cell transfusion was needed in 7 (18.52%) patients. Postoperative pneumonia occurred in 2 of the converted ones, none in the laparoscopic group (4%). The mean postoperative stay was 4 ± 2.11 days (range 3-9 days). One patient developed small hematoma at one 10 mm port site, which required opening of the skin sutures. No patients developed a pancreatic fistula. Rise of platelet counts could be seen up to 12 lakhs/mm3. Steroids and/or azathioprine could be stopped in all patients except one; he still needed low-dose steroids to keep platelet count around 50,000/mm3 at 2.47 years of follow-up. Rise of hemoglobin was seen up to 16gm/dl. The 30-day mortality rate was zero. No other long-term complications were noted.

## 4. Discussion

Advanced laparoscopic surgery has largely been eluded in Nepal due to lack of resources and poverty. Earlier reports have mostly been of laparoscopic cholecystectomy and appendectomy and one of laparoscopic hernia [22–25]. Although laparoscopic splenectomy had been introduced in 2010 in Nepal with a laparoscopic-assisted splenectomy [22], this is the first attempt of contribution to advanced laparoscopic surgery such as splenectomy in largest volume from any center in Nepal. This series of 50 laparoscopic splenectomies by a single surgeon is a step to introduction and establishment of advanced laparoscopic surgery in the region.

Regarding the epidemiology of the diseases for which splenectomy was done, beta-thalassemia trait is the most common hemoglobinopathy in Nepal, hemoglobinopathy being found in 6.66% of the normal population in a screening study in Nepal [26]. Though, in our series, hereditary spherocytosis is the most common cause of anemia for which splenectomy was done. Other than hemoglobinopathies, ITP is also a common blood disorder in Nepalese population and is frequently seen in young women [27]. Poudyal et al. have studied azathioprine as second-line modality for ITP patients; we have studied in those patients who were both azathioprine and steroid refractory/dependent [27]. Shrestha et al. have established open splenectomy to have significant response for these patients before; we have ventured via minimally invasive approach [28]. Most patients due to their inability to afford other expensive options as IVIG and Rituximab accept splenectomy relatively readily.

Splenectomy is not without morbidity and mortalities, and laparoscopic splenectomy is one of the advanced laparoscopic surgeries. We always had an inhibition as whether we would be successful in stepping up from basic laparoscopic surgery. However, we dared to progress, with acquirement of some expensive resources like ultrasonic energy. And here we are, with acceptable morbidity rates of 16% and zero mortality, which is comparable to other studies in the literature [2, 5, 7–10]. However, these resources are still far from ideal as in developed countries, like the endocatch bag, morcellators, and endostaplers which add to the cost of the surgeries, though their use has been reported to decrease the operative time. Ultrasonic energy could be used when the hand shears were available; in about half of all cases, they were not available and dissection was done with the help of monopolar and bipolar diathermy. In addition, the mean operating time of 130 min compares favorably and the mean hospital stay of 7 days is comparable to the majority of other series [11–15]. The conversion rate to open splenectomy was 10% for this series, again comparing favorably to results in the literature [2–6, 9, 10, 14, 15, 18, 19].

There are two approaches to laparoscopic splenectomy: anterior and lateral [3]. We learnt the lateral approach; we still have not attempted laparoscopy in very large spleens (which reach below the umbilicus), in which the anterior approach is more appropriate. In lateral approach the large spleen hanging would obliterate the operative space. Laparoscopic splenectomy for such massive splenomegaly still remains controversial and has traditionally been contraindicated [7]. Data in the literature have demonstrated an increased risk of hemorrhage, morbidity, and conversion to open procedure [29–31]. However, it is well proven from literature that laparoscopic splenectomy is now universally accepted due to low postsurgical pain, hospital stay, low incidence of postoperative pulmonary complications, and wound related complications. Like other solid organs laparoscopy procedures, it possesses specific technical challenges like lack of tactile feedback, difficult assessment of accessory spleens, need of advanced energy sources like harmonic scalpel and endostapler because of inherent possibility of bleeding especially in low platelet counts, and finally the technically demanding manipulation for removal of spleen without breaking the capsule of the spleen [32–34]. All these reasons make this a technically difficult procedure with steep learning curve. However, these can be overcome with continuing experience of the surgeon and persistent perseverance towards continuation of the procedure.

## 5. Conclusion

This case series is one of a series of totally laparoscopic splenectomy in a developing country, which could be successfully contemplated in patients with hematological diseases, especially if the spleen is normal or mildly enlarged. Absence of ideal resources has not limited our progress in minimal access approach. This initial report highlights the safety of laparoscopic splenectomy and expands the horizons of laparoscopic splenectomy and advanced laparoscopic surgery in a developing country like ours.

## Figures and Tables

**Table 1:** Operative characteristics.

	Mean	Median	Standard deviation	Range
Operating time (in min)	130	130	49	108-224
Blood loss (in ml)	456	253	120	Negligible to 1600
Time of orally allowing (in hrs)	8	8	3.2	6-24
Total postoperative stay (in days)	4	4	2.1	3-9
Total hospital stay (in days)	6	7	2.52	4-12

**Table 2:** Postoperative complications.

	Complications	n	%
1	Mortality	0	0
2	Pneumonia	2	4
3	Splenic and portal vein thrombosis	1	4
4	Subcutaneous hematoma	1	2
5.	Intraoperative hemorrhage	5	10

## Data Availability

The datasets used and/or analyzed during the current study are available from the corresponding author on reasonable request.

## Abbreviations

mm: Millimeter
Fr: French
ml: Milliliter
ITP: Idiopathic thrombocytopenic purpura.
